# A Limited International Intercomparison of Responsivity Scales at Fiber Optic Wavelengths

**DOI:** 10.6028/jres.096.011

**Published:** 1991

**Authors:** R. L. Gallawa, J. L. Gardner, D. H. Nettleton, K. D. Stock, T. H. Ward, Xiaoyu Li

**Affiliations:** National Institute of Standards and Technology, Boulder, CO 80303; National Measurement Laboratory, Lindfield, Australia; National Physical Laboratory, Teddington, Middlesex, United Kingdom; Physikalisch-Technische Bundesanstalt, Braunschweig, Germany; National Physical Laboratory, Teddington, Middlesex, United Kingdom; National Institute of Standards and Technology, Boulder, CO 80303

**Keywords:** germanium detectors, international intercomparison, national standards, optical communications, optical detectors, optical power, responsivity

## Abstract

We report here on a recent limited international intercomparison of responsivity scales at wavelengths of interest to the optical communications community. Participants in the comparison were the national laboratories in the United States, the United Kingdom, Germany, and Australia. The wavelengths tested were 1300 and 1550 nm. Data taken at 850 nm are only briefly discussed. The disagreement between the national laboratories’ responsivity scale is comfortably within the uncertainty claimed by each laboratory.

## 1. Introduction

The precise measurement of optical power is of fundamental importance to the proper performance of modern optical communications systems. Power measurements should therefore be traceable to national standards and national responsivity scales should be in reasonable agreement between countries [1]. There have been informal tests of this agreement from time to time but, until now, a formal intercomparison has not been carried out.

We report here on a limited intercomparison that was designed to determine the level of agreement between the responsivity scales of four national standards laboratories whose responsibilities include the calibration of optical power meters for the optical communication community. To that end, we have intercompared the responsivity at 1300 and 1550 nm. The participants were the National Physical Laboratory (NPL), United Kingdom, the Physikalisch-Technische Bundesanstalt (PTB), Germany, the Commonwealth Scientific and Industrial Research Organization (CSIRO), Australia, and the National Institute of Standards and Technology (NIST), USA. In presenting the results, we will refer to these laboratories anonymously as Laboratories A, B, C, and D.

## 2. Background

Various limited round robin experiments have been conducted [2] to determine how best to improve metrology service to the optical community and to understand the sources of error in power measurements [3], [4]. As sources of calibration and measurement error become better understood [5] the uncertainty reported by vendors becomes more dependent on the uncertainty of scales of the national standards laboratories. While it was expected that the agreement between these scales is good, the international nature of the optical communications industry has suggested that a substantiation of this agreement was needed.

The Comite International des Poids et Mesures (CIPM) recognized this need and recommended an international intercomparison of responsivity scales at wavelengths of interest to the optical fiber community. The intercomparison that was to be undertaken as a result of that recommendation is quite ambitious, involving a total of fourteen national laboratories. It seemed prudent, therefore, to take preliminary and precautionary steps to minimize the potential for error and to provide guidance for this ambitious undertaking. This preliminary step took the form of a limited international intercomparison, a pilot study between a limited number of participants. The results of that preliminary intercomparison are reported here. The global intercomparison, involving the 14 laboratories, will be reported in due course.

The purpose of the pilot study was to assess the robustness of the transfer standard and identify problem areas and potential sources of error, and thus to guide the more global experiment. In this experiment, each of the participating laboratories calibrated two detectors at the two wavelengths of most interest, in each case using a power level that gives good signal-to-noise ratio and is commensurate with conditions encountered in the field. Additional measurement conditions are given later.

## 3. Measurement Conditions

Specially selected commercial germanium detectors without coolers and of 5 mm diameter were chosen for the intercomparison. They were selected in advance for linearity, spatial uniformity, and shunt resistance. Launch onto the detector was by open beam with half-angle divergence not exceeding 10 degrees. Incident power of 10 to 50 µW was in a 3 mm diameter spot and centrally positioned to avoid edge effects. The detectors have a window but not an aperture. Measurements were made at 1300 and 1550 nm. A preamplifier was circulated with the diodes.

## 4. Procedure

All reasonable precautions were taken to protect the detectors from the environment during the course of this test. The detectors were handled with reasonable care and shipped in a special shipping carton. The detectors traveled by commercial air transport.

### 4.1 National Laboratory Procedures

#### 4.1.1 NPL

The instrumentation used at NPL is a spectroradiometer comprised of a high stability tungsten filament lamp imaged with a concave mirror onto the entrance slit of a double grating monochromator. The radiation from the monochromator is imaged via an intermediate field stop onto the center of the germanium photodiodes using another concave mirror. The image is circular with a diameter of 3 ± 0.2 mm and is centered to within 0.2 mm. The incident radiation is within 6 degrees of the normal to the detector surface. The spectral bandwidth is 6 nm. The detector electric current output is recorded using a digital voltmeter with a high quality current-to-voltage converter which has an input offset voltage of less than 2 µV. The detectors are compared directly with NPL standards using a translation slide. The spectroradiometer and data collection are under computer control. Laboratory temperature was 20–21 °C and photocurrent was between 0.5 and 8 µA.

The traceability route at NPL is as follows:

1. Optical power in a laser beam at 800 nm is measured with the NPL cryogenic radiometer, the NPL primary standard for optical power measurements.
2. The data are converted to absolute spectral responsivity of silicon photodiodes at 800 nm using the same laser beam.
3. Conversion is then to absolute spectral responsivity of the germanium photodiodes at 900 nm using dispersed radiation and the NPL relative spectral responsivity scale maintained with a vacuum thermopile. The relative spectral responsivity of this detector has been calibrated from 240 nm to 1 µm from reflectance measurements.
4. Conversion of the data are then to the absolute spectral responsivity of the germanium photodiodes at 1300 and 1550 nm using dispersed radiation and the NPL near infrared relative spectral responsivity scale established with pyroelectric detectors. The relative spectral responsivity of this detector has been calibrated from 800 nm to 1700 nm from reflectance measurements.

#### 4.1.2 PTB

1. The absolute calibration is at λ, = 1047 nm. The primary standard is an electrically calibrated thermal cone detector [6]. The calibration is transferred to a secondary thermopile standard at λ. = 1047 nm (Nd:YLF-laser). Transfer is then to the germanium diodes (λ. = 1047 nm) using a laser spot of 3 mm diameter. The nonlinearity and the temperature coefficient of the thermopile are accounted for [7]. The spot position, size and homogeneity are monitored using a CCD camera which is sensitive at 1047 nm. The power level is from 820 to 880 µW.
2. To determine the calibrations at 1300 and 1550 nm relative to 1047 nm, the relative spectral measurements are performed with a 0.3 m flintglass prism double monochromator [8]. The secondary standard for the relative measurements is another thermopile whose relative responsivity is constant within ±5×10^−4^, as determined by a comparison with a thermal cavity detector [9]. The current levels ranged from 0.4 to 2.7 µA and the spectral bandwidths from 9 to 16 nm. Laboratory temperature was 22 ±3 °C.

#### 4.1.3 CSIRO

The primary standard for radiometric power (and hence responsivity) is determined at 633 nm xising inversion layer silicon photodiodes operated under conditions of unity internal quantum yield. The technique is described in reference [10]. An integrating sphere reflectometer [11] is now used. A gold-black bolometer is used [12] as a standard of relative spectral responsivity. For these measurements, a 3 mm diameter InGaAs photodiode is used as a working standard; its absolute responsivity was measured at 633 nm against the inversion layer photodiodes. The InGaAs photodiode was then compared with the bolometer at 633, 1300, and 1550 nm. Finally, the test detectors are compared to the InGaAs detector. A 0.5 m monochromator with prism predisperser is used for wavelength selection with a bandwidth of 8 nm. Supplementary filters are required at 1300 and 1550 nm to reduce the second-order to negligible levels. Dual concave spherical mirrors direct the light from the monochromator onto the test and reference detector in turn, rotating the second mirror symmetrically about the beam from the first. Apertures placed between the mirrors, both filled, are imaged with different magnifications, onto the two detectors being compared. Measurements were made at a laboratory temperature of 21 ± 1 °C. The diodes were temperature controlled at 22 ± 1 °C.

#### 4.1.4 NIST

The calibration of optical power at NIST is based on a standard reference instrument called the C-series calorimeter. Details are given in reference [13]. The calorimeter is a national reference standard for measuring absolute energy or power of cw laser sources over a wide range of wavelengths. Infrared laser sources and calibrated beamsplitter measurement systems are used to compare an electrically calibrated pyroelectric radiometer (ECPR) to the C-series calorimeter. The calibrated ECPR is then used as a laboratory standard for the calibration of optical power meters at the wavelengths of interest to the fiber community.

The ECPR was selected as the reference standard because it has a large absorbing surface (about 8 mm diameter), high absorptivity over a wide range of wavelengths and angles of incidence, and it is spectrally quite flat over the wavelength range of interest.

Stabilized laser diode sources provide laser power at the desired wavelengths through a fiber pigtail that traverses a mandrel wrap intended to eliminate cladding modes and induce power stability. The mandrel wrap also produces a beam that has a uniform spatial distribution. The beam that exits from the fiber is collimated by lenses whose focal lengths are such that the collimated beam has nominal diameter of 3 mm. The laser sources, associated fibers, and lenses are configured on a computer controlled positioning table.

The ECPR and the meter to be calibrated (the test meter or detector) are placed in close proximity on the test bench and oriented to allow the beam to be incident, in turn, on the ECPR and then on the test meter, each of which is allowed to reach steady state before a number of readings are taken and averaged. This comparison is repeated ten to twenty times. A computer is used to control the entire process and to calculate the results. The comparison is facilitated by moving the source, pigtail, mandrel wrap, and lenses, as one unit, thus avoiding the variations that might result when the fiber or associated paraphernalia are moved, even slightly. Measurements were at 22 ± 1 °C.

## 5. Calibration Results

**Figure 1:**
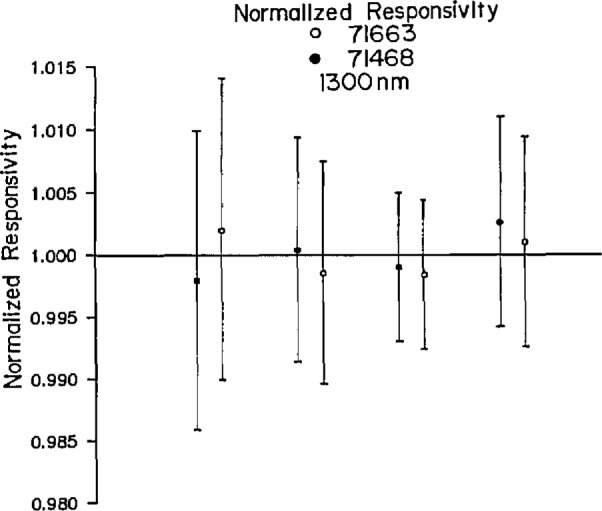
Normalized responsivity at 1300 nm, according to laboratory (Laboratories A, B, C, and D, positioned left to right in the figure) and detector.

**Figure 2:**
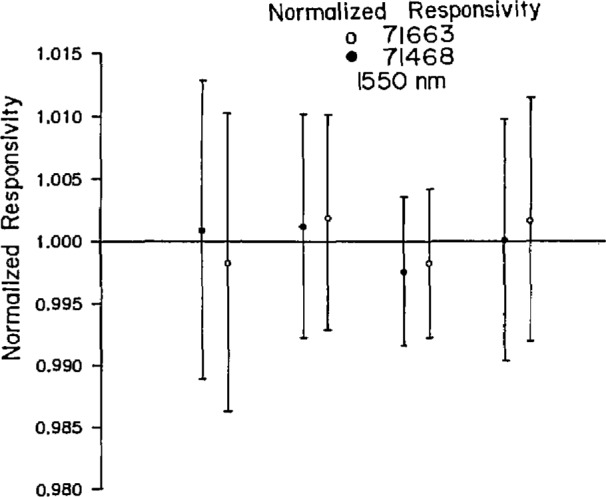
Normalized responsivity at 1550 nm, according to laboratory (Laboratories A, B, C, and D, positioned left to right in the figure) and detector.

The results of the calibration test are given in table 1 and in figures 1 and 2, which give the data in graphical form for the two wavelengths. The figures show the normalized data according to laboratory (Laboratories A, B, C, and D, positioned left to right in the figures), and detector number. The data are normalized to the average value for all laboratories in each figure (i.e., at each wavelength). The data are presented anonymously because these calibrations were intended primarily to determine the feasibility of using germanium photodiodes as transfer standards and to circumscribe the problems associated with an international intercomparison.

Table 1 shows that the maximum difference between any two of the measured responsivities is always less than 0.5%. The maximum deviation by any one laboratory from the average of the calibration factors is 0.36% in three of the four measurements. All data are within the uncertainties of each of the national laboratories.

There is no indication of systematic differences in the data. One laboratory is not consistently lower or consistently higher than the others, thus indicating that random errors are being encountered. In fact, each laboratory has exactly one entry in the column of table 1 that is labeled “maximum deviation from the average.” That is, each of the laboratories is furthest from average value exactly once.

It is encouraging to note that the agreement in the calibration is slightly better at 1550 nm (the likely wavelength of future systems) than it is at 1300 nm.

Three of the four laboratories took responsivity data at 850 and 800 nm as well. Since gemanium detectors were used as transfer standards, there was more variability amongst the laboratories than at the long wavelengths, but this can be attributed to higher reflectances, aging [1], and inhomogeneities [14] at the shorter wavelengths. The maximum discrepancy between any two laboratories was 1.2% at 850 nm using detector 71663. We conclude that germanium detectors are probably not suitable at 850 nm; silicon is more appropriate and should be used as transfer standards for the shorter wavelengths. Data for the short wavelengths are not included here because the global international intercomparison will not use the shorter wavelengths. Furthermore, germanium is not the material of choice at the 850 nm window.

Table 1 includes the uncertainty reported by each laboratory. The uncertainty is determined, in each case, according to the rules recommended by BIPM [15]. Each laboratory included Type A uncertainty and Type B uncertainty, which correspond, respectively, to what was once called random and systematic uncertainties (or errors, as they are sometimes called). Type A uncertainties are taken to be normally distributed whereas Type B uncertainties are assumed to be uniformally distributed (sometimes referred to as a rectangular or “top hat” distribution). The width of the Type B uncertainties distribution is difficult to determine in most cases, since they are not based on statistical methods. Each of the participating laboratories determines the width for each contributor according to his own experience.

These and other nuances of the statement of uncertainties have received a lot of attention by the BIPM and the details are beyond the scope of this paper. For our purposes, suffice it to say that each of the participating laboratories reports a total uncertainty, given in the table, that is composed of Type A and Type B uncertainties, combined in quadrature and multiplied by a constant (in our case the constant is 3) to determine a number that is thought of as the uncertainty in the measurement. If the constant is 3, the uncertainty is stated as being at the three sigma (or the 99%) confidence level. It is this uncertainty that is given in table 1.

## 6. Conclusions

There was good agreement between the responsivity scales of the national laboratories that participated in this intercomparison. Only the wavelengths of interest to the optical fiber community were considered. The data show an encouraging consistency, showing 0.36% maximum difference between any two laboratories. The maximum deviation of any one laboratory from the average was only 0.19 to 0.26%. It seems clear that a transfer standard calibrated by any one of these four laboratories could be used interchangeably with one calibrated by any one of the other three laboratories without loss of accuracy, provided the standards are well characterized and carefully selected.

From these results, we conclude that the germanium detectors that were used here are robust enough to survive the rigors of international comparison when suitable care is taken. The experience gained here will guide us in proceeding to the more global round robin intercomparison that is now underway. In that intercomparison, approximately 14 national laboratories will participate using similar but different detectors.

## Figures and Tables

**Table 1:** International intercomparison spectral responsivity (A/W)

1300 nm							
Uncertainty (%)	Lab A 1.2	LabB 0.9	LabC 0.6	LabD 0.84	Av.	Max, dev. from av. (%)	Max. diff. (%)
Det. 71468	0.6869	0.6886	0.6876	0.6901	0.6883	0.26	0.46
Det. 71663	0.6898	0.6874	0.6873	0.6891	0.6884	0.20	0.36

1550 nm							
Uncertainty (%)	Lab A 1.2	LabB 0.9	LabC 0.6	LabD 0.97	Av.	Max. dev. from av. (%)	Max. diff. (%)
Det. 71468	0.9055	0.9058	0.9025	0.9048	0.9047	0.24	0.36
Det. 71663	0.9061	0.9094	0.9061	0.9092	0.9077	0.19	0.36

a Uncertainty is given at three sigma or 99% confidence level.
